# Diagnosis and Management of Aortic Dissection in a Pediatric Patient With Turner Syndrome: A Simulation

**DOI:** 10.7759/cureus.41725

**Published:** 2023-07-11

**Authors:** Megan M Attridge, Kali A Hopkins, Karen Mangold, Wendy J Brickman, Sheetal R Patel

**Affiliations:** 1 Pediatric Emergency Medicine, Ann and Robert H. Lurie Children's Hospital of Chicago, Chicago, USA; 2 Pediatrics, Northwestern University Feinberg School of Medicine, Chicago, USA; 3 Adult Congenital Cardiology, Division of Cardiology, Mount Sinai Cardiovascular Institute, Icahn School of Medicine at Mount Sinai, New York, USA; 4 Pediatric Endocrinology, Ann and Robert H. Lurie Children's Hospital of Chicago, Chicago, USA; 5 Pediatric Cardiology, Ann and Robert H. Lurie Children's Hospital of Chicago, Chicago, USA

**Keywords:** pediatric clinical cardiology, chest pain in the young, simulation medicine, pediatric emergency & acute care, turner syndrome, aortic dissection management, aortic dissection diagnosis, aortic dissection, simulation training

## Abstract

Aortic dissection is exceedingly rare in the pediatric population. However, it is much more common among children and adolescents with certain underlying syndromes, including Turner syndrome. Furthermore, aortic dissection carries significant mortality without prompt diagnosis and management. Therefore, pediatric emergency providers should know how to recognize and treat pediatric aortic dissection in a patient with Turner syndrome. We designed this simulation for pediatric emergency medicine fellows. A simulated adolescent female patient with a known history of Turner syndrome presents with chest pain, tachycardia, and hypertension. Participants must order and interpret the appropriate diagnostics, diagnose aortic dissection, and manage aortic dissection adequately. This simulation was completed by six pediatric emergency medicine fellows and one pediatric resident. After completing the simulation, six participants (85.7%) provided anonymous feedback on a five-point Likert scale (one = strongly disagree, five = strongly agree). Feedback was positive, and participants agreed that the case content was relevant to their clinical practice and that the event will improve their clinical practice. This simulation encourages participants to recognize and manage pediatric aortic dissection in patients with Turner syndrome. Participants felt that the simulation was relevant and will improve their clinical practice.

## Introduction

Turner syndrome is a relatively common genetic condition, characterized by complete or partial loss of an X chromosome, affecting one in 2,500 live female births [1-3]. Typical features of Turner syndrome include short stature, primary amenorrhea, hypogonadism resulting in lack of or delayed sexual development, and increased risk of cardiovascular malformations [1,4,5]. Cardiovascular manifestations are the largest contributors to the early mortality seen in this population. The rare but life-threatening complication of aortic dissection accounts for up to eight percent of all deaths in this population [5,6].

Aortic dissection is extremely rare in the pediatric and young adult population but has a high rate of mortality of up to 22% [7]. Predisposing factors include cardiovascular manifestations that are more common in individuals with Turner syndrome, including bicuspid aortic valve, hypertension, aortic dilation, and coarctation of the aorta [5,6]. Individuals with Turner syndrome who become pregnant are at further increased risk for aortic dissection [8]. Additionally, some patients with Turner syndrome develop aortic dilatation without classic predisposing factors indicating underlying vasculopathy that places all patients with Turner syndrome at risk even without other risk factors [9-11]. Individuals with Turner syndrome are approximately six to 12 times more likely to develop an aortic dissection and present at a much younger age with aortic dissection compared to the general population [4,12]. Diagnosis is often delayed due to the rarity of aortic dissection in general and the younger age of presentation in the Turner syndrome population [4].

The most common symptom at the time of aortic dissection presentation is chest pain with the location dependent upon the location of the dissection [13]. The majority of aortic dissections in patients with Turner syndrome occur in the ascending aorta [4], which is usually characterized by abrupt, severe, sharp, or tearing chest pain with or without radiation to the back. End-organ involvement can lead to cerebrovascular accidents, myocardial infarction, acute aortic regurgitation, cardiac tamponade, or mesenteric ischemia. Physical examination can reveal tachycardia, a new murmur due to aortic regurgitation, pulse deficits, and abnormal blood pressure [13].

According to guidelines from the American Heart Association in 2010, the initial evaluation when there is a high suspicion for aortic dissection should include an electrocardiogram and a rapid imaging modality for assessing the aorta. Possible imaging studies include chest computed tomography which is rapid and more readily available compared to other tests such as transesophageal echocardiogram, or chest magnetic resonance imaging [13]. The choice of imaging study is ultimately based on the patient’s stability and the institution’s capabilities. While not diagnostic, additional labs and imaging may help lead to the diagnosis of aortic dissection and prompt diagnostic imaging. Labs may demonstrate elevated D-dimer, leukocytosis, troponin elevation, or acute kidney injury [13-15]. A chest X-ray can show a widening of the aortic silhouette or abnormal aortic contour [16]. Point-of-care ultrasound can reveal aortic regurgitation, pericardial effusion, intimal flap, or aortic root dilation [17]. Management of acute aortic dissection focuses on reducing aortic wall stress by controlling tachycardia and hypertension with intravenous beta-blockade, and simultaneous urgent consultation with cardiovascular surgeons for consideration of emergent surgical repair; pain may be controlled with intravenous opioids [13].

Because of the rarity of aortic dissection in pediatric and adolescent patients, few pediatric providers are experienced in managing aortic dissection. Clinicians must maintain a high index of suspicion to recognize this life-threatening condition while keeping in mind the risk factors associated with dissection, including Turner syndrome [4,13]. Aortic dissection is also more common in connective tissue disorders such as Marfan and Type IV Ehlers-Danlos syndromes, so knowledge of how to diagnose and manage aortic dissection can be helpful in treating those patients as well [7]. It is imperative to diagnose rapidly, begin treatment as soon as the diagnosis is made, and communicate effectively with multiple disciplines, including radiologists, cardiologists, and surgeons. In a literature search for simulations, one article focused on the simulation of diagnosis and management of aortic dissection with none in the pediatric population and none in patients with Turner syndrome [18]. We, therefore, designed a simulation focused on the diagnosis and management of aortic dissection in an adolescent with Turner syndrome.

This work was previously presented as a poster abstract at the Northwestern Feinberg School of Medicine Medical Education Day on September 30th, 2022.

## Technical report

Learning objectives

By the end of this simulation, participants will complete the following learning objectives: 1) Organize a patient-care team. 2) Demonstrate rapid assessment of hemodynamically abnormal pediatric patients. 3) Consider the differential diagnosis of severe chest pain in a pediatric patient. 4) Recognize the increased risk of aortic dissection in patients with Turner syndrome. 5) Order appropriate labs and imaging and notify the appropriate consultants. 6) Diagnose aortic dissection. 7) Obtain appropriate access and administer the proper medical management, including appropriate antihypertensives and pain control.

Methods

This simulation was developed at the request of the family of a woman with Turner syndrome who died in the setting of a delayed diagnosis of aortic dissection. It was developed for pediatric emergency medicine (PEM) fellows, but it is appropriate for emergency medicine residents of all levels of training, senior pediatric residents, and pediatric cardiology trainees. This simulation was a collaboration by specialists from PEM, pediatric cardiology, and pediatric endocrinology. This simulation was approved by the Institutional Review Board at Ann & Robert H. Lurie Children’s Hospital of Chicago. The entire simulation guide and flowsheet are available in Appendix A.

Setting and equipment

**Figure 1:**
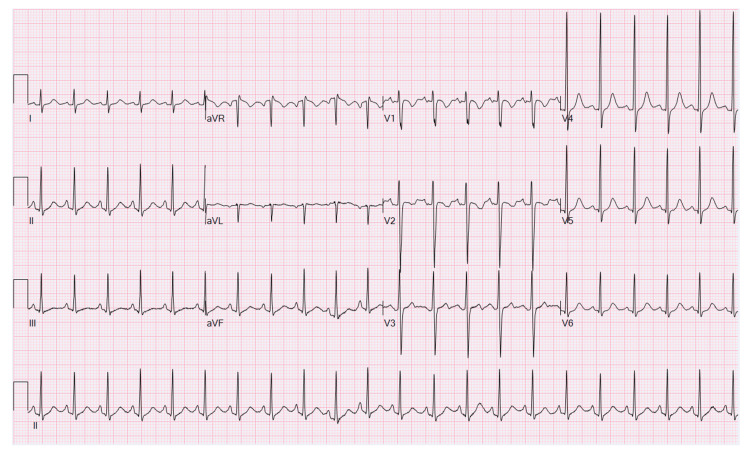
Electrocardiogram This electrocardiogram demonstrates sinus tachycardia. Image credit: Kali A. Hopkins, MD (author).

**Figure 2:**
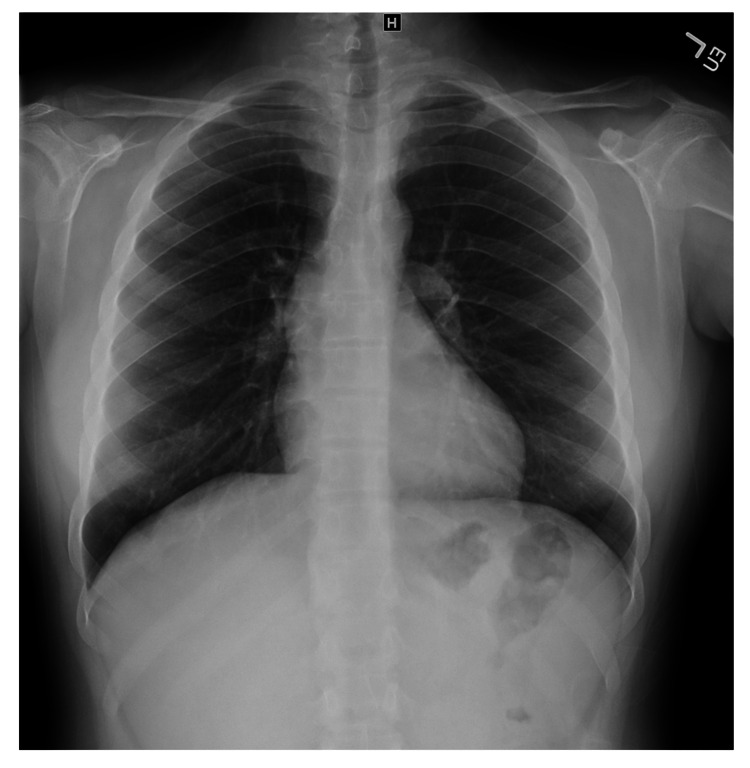
Chest Radiograph This chest radiograph demonstrates a mildly widened mediastinum with prominence of the aortic knob. Image credit: Kali A. Hopkins, MD (author).

**Figure 3:**
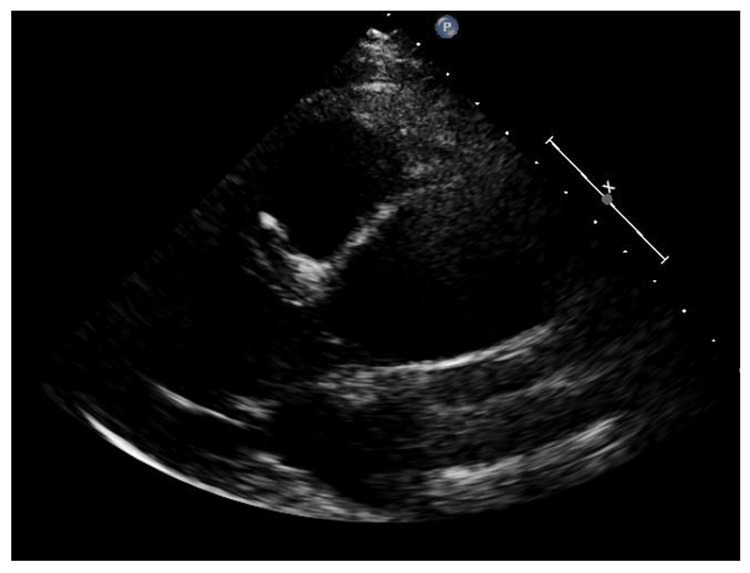
Point-of-Care Ultrasound This point-of-care ultrasound demonstrates ascending aorta dilation. Image credit: Sheetal R. Patel, MD, MSCI (author).

**Figure 4:**
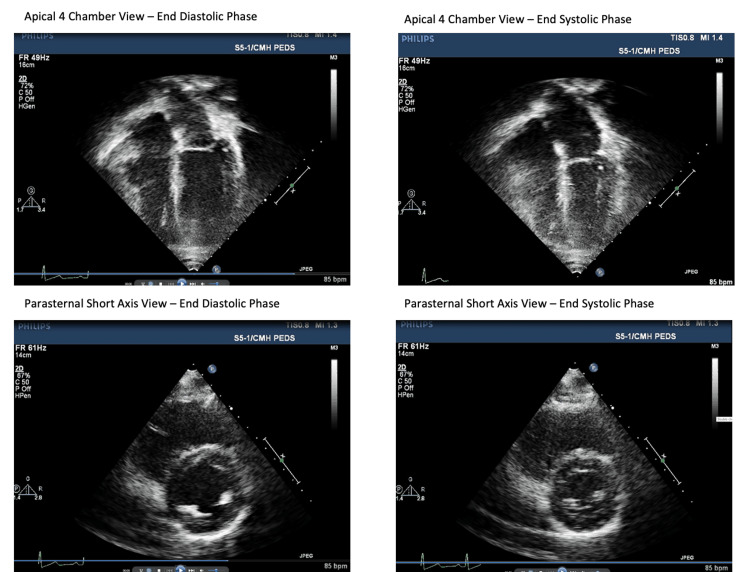
Point-of-Care Ultrasound This point-of-care ultrasound demonstrates a normal function. Image credit: Sheetal R. Patel, MD, MSCI (author).

**Figure 5:**
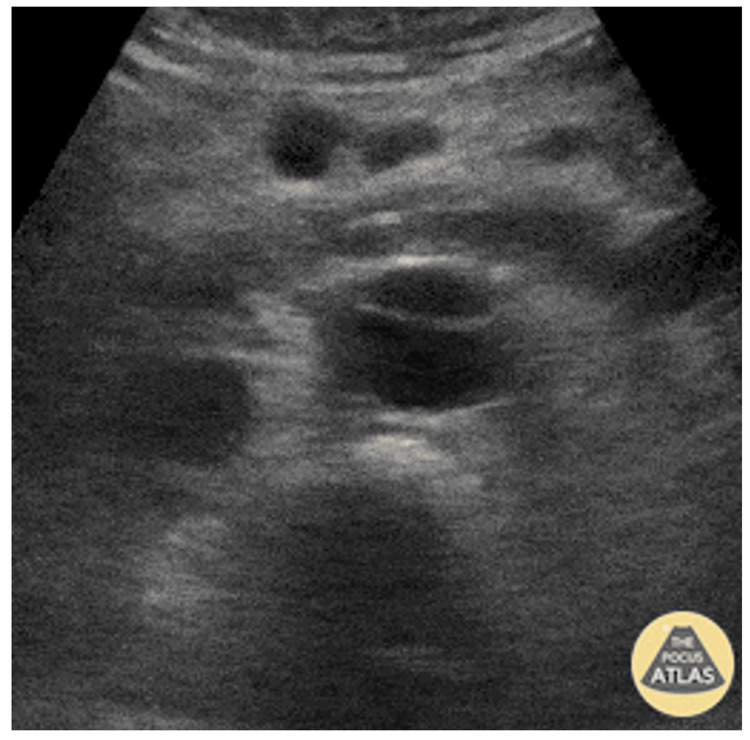
Point-of-Care Ultrasound This point-of-care ultrasound demonstrates an aortic dissection flap in the transverse view. Image by Richard Cunningham, MD, retrieved from The POCUS Atlas: https://www.thepocusatlas.com/aorta/aortic-dissection-flap on 9/15/2022. Content licensed under the Attribution-NonCommercial 4.0 International (CC BY-NC 4.0).

**Figure 6:**
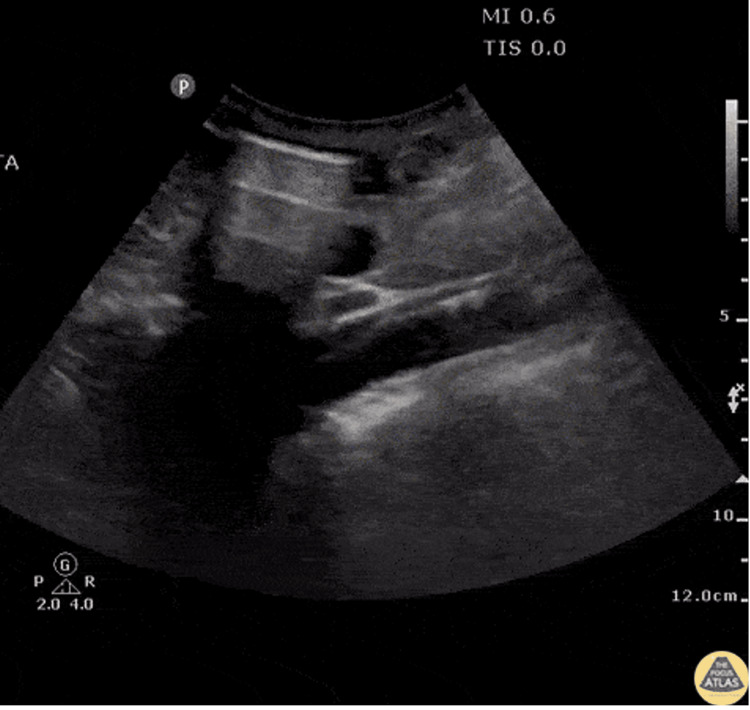
Point-of-Care Ultrasound This point-of-care ultrasound demonstrates an aortic dissection flap in the longitudinal view. Image by Robert Jones, DO, FACEP, retrieved from The POCUS Atlas: https://www.thepocusatlas.com/aorta/aortic-dissection on 9/15/2022. Content licensed under the Attribution-NonCommercial 4.0 International (CC BY-NC 4.0).

This simulation was performed in a procedure room or medical resuscitation bay in the pediatric emergency department. This simulation utilized a female simulator (school age to adolescent or adult size) with capabilities for heart and lung sounds; standard monitoring equipment to assess heart rate, respiratory rate, pulse oximetry, and non-invasive blood pressure; a code cart with typical supplies for intravenous access and fluids, airway management, code medications, syringes, and needles; additional medications including an esmolol infusion, labetalol, nicardipine, hydralazine, fentanyl, and morphine; as well as a bedside ultrasound and electrocardiogram machine. The visual cues available in Appendix B were used to display initial labs (complete blood count, basic metabolic panel, point-of-care venous blood gas with electrolytes, troponin, D-dimer), electrocardiogram (Figure 1, Appendix B ), and imaging results (Figures 2-6, Appendix B; cardiac point-of-care ultrasound images, chest radiograph).

Participants

This simulation was run twice with seven total participants at many levels of training including one senior pediatric resident and six PEM fellows of varied levels of experience from April 2021 through July 2021. PEM fellows participated as part of a mandatory monthly simulation curriculum focused on pediatric emergencies; pediatric resident participation was optional and voluntary. No prerequisite knowledge was required for this case, and there was no assigned pre-reading.

Personnel

Embedded actors included the PEM fellow who designed this simulation, PEM attendings, a pediatric cardiology fellow, and nurse simulation specialists. For each simulation, two embedded actors acted as bedside nurses. They also could provide additional information about the patient’s physical exam findings and other information from the patient's perspective upon participant request. An additional embedded actor was the caregiver of the simulated patient and could provide medical history and events leading up to the presentation. The pediatric cardiology fellow was available as needed to answer consultation calls from the participants. A PEM fellow facilitated the simulation and was available to answer calls from other consultants as required. A simulation technician was responsible for adjusting vital signs throughout the simulation.

Pre-briefing

The PEM fellow facilitator oriented the participants to the simulator, explained the emergency department setting, and introduced a learning contract. Participants were asked to wait outside of the simulation room at the start of the simulation.

Case summary

The acting bedside nurse called participants into the simulated patient’s space to introduce the patient as having chest pain to initiate the simulation. Roles were not assigned before the initiation of the simulation and were decided amongst participants upon initiation of the simulation, which included a team leader, airway, and a physician to perform an initial assessment and obtain a history. The participants were responsible for performing the initial assessment, asking for the patient to be placed on monitors to obtain an initial set of vital signs, and getting a history from the patient and the patient’s caregiver. When asked, the patient revealed she had severe chest pain that radiated to her back, which started immediately before the presentation. Initial vital signs and assessment were notable for tachycardia, hypertension, mild tachypnea with splinting, and normal pulse oximetry on room air. When asked, the facilitator acting as the patient’s caregiver would only reveal the patient’s history of Turner syndrome, bicuspid aortic valve, and hypertension. During this assessment and history taking, vital signs remained abnormal, and the patient continued to complain of pain. The case was discussed with cardiology, and heart rate, pain, and hypertension improved with the initiation of intravenous beta-blockade and intravenous opioids. The appropriate imaging was ordered, and cardiothoracic surgery was consulted.

All facilitators and simulation technicians stayed in the same room as the participants. The pediatric cardiology fellow consultant was also available inside the room and could communicate consultant recommendations as needed over the phone. The simulation took about 15 minutes to complete. Visual cues, including chest radiographs, electrocardiograms, echocardiograms, and laboratory values are available in Appendix B. The critical actions checklist is available in Appendix C.

Debriefing

After completing the simulation, facilitators conducted a debriefing of approximately 35 minutes. The pediatric cardiology fellow who was acting as a cardiology consultant during the simulation was present to answer unanticipated questions about complex cardiac pathophysiology and resuscitation. Debriefing was performed by experienced debriefers focused on providing a supportive and safe environment. Their debrief was styled after the PEARLS Healthcare Debriefing Tool which provides a structured framework to assist debriefing simulations with the goals of improving teamwork and clinical decision-making [19,20]. Initial debriefing questions and prompts, based on the PEARLS Healthcare Debriefing Tool focused on teamwork and clinical decision-making: "What were your first reactions?" "Summarize the case. Did anyone understand the scenario to be different?" "What went well?" and "What would you have done differently?" Additional questions and prompts focused on medical management including: "What do you remember about the clinical characteristics common to patients with Turner syndrome?" "How do you approach the differential diagnosis of chest pain in a patient with Turner Syndrome?" and "How is a diagnosis of aortic dissection made?" and "How is aortic dissection managed?" After debriefing, key learning points about presentation, diagnosis, and management were discussed with participants.

Assessment

The PEM fellow facilitator reviewed the completion of critical actions. A PEM fellow facilitator, the pediatric cardiology fellow, and PEM attending facilitators reviewed these items and provided feedback to participants during the debriefing.

All learners correctly diagnosed aortic dissection, appropriately consulted cardiology for management recommendations, and adequately treated the patient with pain control and antihypertensives. Participants adequately communicated with each other and with the caregiver. Facilitators observed closed-loop communication.

After completing the simulation and debriefing, participants completed an anonymous and optional feedback survey. Participants responded to statements on a five-point Likert scale (one = strongly disagree, five = strongly agree). Statements centered around the simulation’s relevance to clinical practice, adequacy of debriefing, and effect on clinical practice. The feedback form is outlined in Appendix D.

Case evaluations were positive overall. Of the seven participants, six individuals responded to the survey (85.7%). All responding participants “strongly agreed” that the case content was relevant to their clinical practice and sufficiently challenging; that in the simulation they were placed in a role consistent with their professional setting; that in the debriefing learners had opportunities to speak and were listened to, medical issues were addressed, and teamwork and communication issues were addressed; and that the event will improve their clinical practice with mean Likert scores of five.

## Discussion

We developed a novel simulation case of aortic dissection in a patient with Turner syndrome to help participants recognize this rare event that requires early diagnosis and prompt management. We have shown that this simulation is a practical means to provide educational content on managing this unique patient population. Overall participants felt the case content was relevant to their clinical practice and that the simulation would improve their clinical practice.

In this simulation, all participants were able to elicit the history of Turner syndrome and make the correct diagnosis of aortic dissection with assistance from the pediatric cardiology fellow who was appropriately consulted. However, the participants expressed that aortic dissection was not initially on their differential and that they were unaware that patients with Turner syndrome were at increased risk of aortic dissection. Participants were also able to manage aortic dissection appropriately with help from their cardiology fellows. Still, they expressed that they would have had difficulty managing aortic dissection without consultant assistance as it is an infrequent diagnosis in the general pediatric population. These knowledge gaps highlight the importance of further education and training in this life-threatening diagnosis. Learners indicated that the simulation was valuable as they previously had no exposure to aortic dissection in pediatric patients.

This case utilizes standard simulation materials and materials available in standard emergency department resuscitation rooms. This simulation topic is infrequently encountered and is appropriate for all PEM fellows' trainee levels. While this case was built for PEM fellows, it is also suitable for emergency medicine residents who may encounter this clinical scenario in their practice. While emergency medicine residents more frequently encounter aortic dissection in the older populations they treat, they may not consider the diagnosis of aortic dissection in the differential of a child or young adult with chest pain. They may be unfamiliar with its association with Turner syndrome.

One limitation of this simulation is the reliance on multiple actors. However, this could be mitigated by having the patient give her own history instead of an actor being a parent, having the facilitator act as the cardiology consultant, or having the simulation technologist or embedded nurse announce any changes to the patient’s physical examination. Another limitation is the primary outcome being a subjective survey. Future directions could include a validated assessment tool, such as a checklist, or repetition of the simulation to assess retention. Additionally, the learning audience could be expanded to include emergency medicine residents and cardiology fellows as knowledge would be pertinent to their training. Finally, as mentioned previously, aortic dissection is exceedingly rare and requires a high index of suspicion when considering a differential diagnosis for pediatric chest pain; a provider may not encounter this diagnosis during their career. However, the purpose of this simulation is to prepare providers for prompt diagnosis and management should they encounter this rare event.

## Conclusions

Aortic dissection, while rare, is more common among patients with Turner syndrome. Overall, aortic dissection is not a commonly encountered medical diagnosis for pediatric emergency medicine providers, and other pediatric acute care providers, who are therefore unlikely to feel comfortable with the diagnosis and management of aortic dissection. This simulation encourages participants to recognize and manage pediatric aortic dissection in patients with Turner syndrome. Participants felt that the case content was relevant to their clinical practice and sufficiently challenging and that the simulation will improve their clinical practice. This technical report provides facilitators with instructions and materials to implement a simulation about aortic dissection in Turner syndrome at their own institution.

## Appendices

Appendix A: simulation guide

**Table 1:** Simulation Guide RN: Registered Nurse; MD: Physician; HR: Heart Rate; T: Temperature; C: Celcius; BP: Blood Pressure; mmHg: Millimeters of Mercury; RR: Respiratory Rate; SpO2: Pulse Oximetry; SAMPLE History: Signs & Symptoms, Allergies, Medications, Past Medical History, Last Menstrual Cycle/Last Meal, Events Leading Up To Presentation; GCS: Glasgow Coma Scale; POC: Point-of-Care; VBG: Venous Blood Gas; CBC: Complete Blood Count; BMP: Basic Metabolic Panel; IV: Intravenous; ECG: Electrocardiogram; CXR: Chest Radiograph; POCUS: Point-of-Care Ultrasound; CTA: Computed Tomography Angiography; CT: Cardiothoracic; HCG: Pregnancy Test; OR: Operating Room; CICU: Cardiac Intensive Care Unit

Simulation Guide			
Introduction			
Patient Age and Sex: 12-year-old female; Patient Weight: 30 kilograms; Chief Complaint: severe chest pain Description of Case: A 12-year-old female is presenting to the emergency department with sudden onset severe chest pain, that started immediately prior to presentation. She is found to have a history of Turner syndrome, and acute aortic dissection requiring the appropriate diagnostic workup and prompt management including hypertension and pain management as well as emergent surgical referral.			
Primary Learning Objectives			
(1) Organize a patient-care team. (2) Demonstrate rapid assessment of hemodynamically abnormal pediatric patients. (3) Consider the differential diagnosis of severe chest pain in a pediatric patient. (4) Recognize the increased risk of aortic dissection in patients with Turner syndrome. (5) Order appropriate labs and imaging and notify the appropriate consultants. (6) Diagnose aortic dissection. (7) Obtain appropriate access and administer the proper medical management, including appropriate antihypertensives and pain control.			
Actors and Roles			
(1) Bedside nurse (RN) #1 can be played by a facilitator or embedded participant. (2) Bedside nurse (RN) #2 can be played by a facilitator or embedded participant. (3) Doctor #1: Team Leader Physician (MD). (4) Doctor #2: Survey MD. (5) Doctor #3: Airway MD. (6) Doctor #4: Family Correspondent (optional, this role can be combined with #1, #2, or #3). (7) Instructor #1: Simulation leader and debriefer, if a 3rd instructor is not available, the facilitator can play the role of the parent as well. (8) Instructor #2: Cardiology consultant, ideally played by a cardiology fellow, but can be played by a facilitator or embedded participant. (9) Instructor #3: If a 3rd instructor is available, they may be cast as a “parent” and assist with debriefing.			
Initial Presentation			
Vital Signs: Heart Rate (HR) = 120, sinus tachycardia on the monitor; Temperature (T) = 37 degrees Celsius (C); Blood Pressure (BP) = 150/90 mmHg; Respiratory Rate (RR) = 26; Pulse Oximetry (SpO2) = 95% on room air; Overall Appearance: A short female who looks younger than her stated age. She appears very uncomfortable and distressed due to pain. History of Present Illness: A 12-year-old female with severe chest pain which started suddenly, immediately prior to presentation. She is feeling short of breath due to the pain. Her parents say she has been “healthy” before today.			
Additional History (if asked)			
SAMPLE history: Signs & Symptoms: Was in her usual state of health when she developed severe tearing chest pain that radiates to her back. The pain is worse with exertion, and she feels short of breath due to the pain. Allergies: none. Medications: lisinopril, estrogen, growth hormone. Past medical history: History of Turner syndrome and hypertension. Takes estrogen to induce puberty and growth hormone to help with short stature. Lisinopril is for her hypertension. Last menstrual cycle, last oral intake: Has never had a period. She ate a normal breakfast a few hours prior to arrival. Events leading up to the event: Pain started out of nowhere. She was not doing anything special. No trauma. No recent illness. Family History: not significant.			
Primary Survey (if performed)			
Airway intact (talking). Breathing is clear to auscultation bilaterally, mild tachypnea and splinting. Circulation notable for weak pulses, capillary refill is 2 seconds. Disability assessment is notable for a Glasgow Coma Scale (GCS) 15; Pupils are 3 millimeters, equal and reactive. Exposure (below).			
Secondary Assessment (if performed)			
The patient is small for stated age; moaning and complaining of pain, her head is normocephalic and atraumatic; she is tearful with moist mucous membranes; her neck is supple with the full range of motion; her lungs are clear to auscultation bilaterally, with no stridor, no wheeze; she has mild tachypnea and splinting due to pain; she is tachycardic, with a regular rhythm, no murmur, no gallop; her extremity pulses are weak; she has no edema, and her capillary refill is 2 seconds; her abdomen is soft, non-distended, non-tender, no masses; she is alert, pupils are equal and reactive to light 3 millimeters-->2 millimeters, extraocular muscle function is normal, moves all extremities well, normal tone, able to speak in full sentences despite discomfort; her skin is warm and dry, with no rashes; her genital exam is normal external female genitalia; Tanner stage II.			
Intervention/Time Point	Facilitator Actions	Learner Actions	Additional Information and Prompts
0-5 minutes	RN notifies the team that they are concerned about their patient who is in a lot of pain. They ask MD and team to evaluate. They can provide a brief history from the history (above).	Establish roles. Perform a primary survey (see above). Place on continuous cardiopulmonary monitoring. Establish intravascular (IV) access. Obtain SAMPLE history from the parent (see above). Request labs with vascular access placement: Point-of-care (POC) venous blood gas (VBG) with electrolytes, D-dimer, complete blood count (CBC), basic metabolic panel (BMP), troponin (these can be requested but are pending after request).	Facilitators can provide information about the primary survey, SAMPLE history as they are asked and performed. Labs are not yet available. If labs are not ordered with vascular access placement, embedded RN can ask MD team "would you like any labs with IV placement?"
6-10 minutes	Parent and RN express concern that she is in significant pain.	Request intravenous (IV) opioid pain medication. Provide a summary statement (pertinent positives from SAMPLE history and primary survey). Complete the secondary survey (see above). Formulate a differential diagnosis for pediatric chest pain (could include myocardial infarction/ischemia, pneumothorax, pneumomediastinum, pulmonary embolism, arrhythmia, pericarditis or myocarditis, anomalous coronary arteries, peptic ulcer disease, aortic dissection). Asks if any labs have resulted. Request bedside electrocardiogram (ECG), chest radiograph (CXR), and cardiac point-of-care ultrasound (POCUS). Call cardiology consult.	Facilitators can provide information about the secondary survey as it is performed. The POC VBG is the only lab available at this time (see Table 2) If cardiology consult not called, embedded parent can mention “I should mention she also sees a cardiologist - I’m not sure why, I think everything is fine, do you think it could be her heart? Should we call the cardiologist?” If lab results are not requested, embedded RN can make MD team aware that some have returned.
11-15 minutes	Update MD that the patient's pain has improved slightly. Update MD that ECG, CXR, and cardiac POCUS images are available and present visual cues. Cardiology consultant discusses the case with MD team. They request a summary of presenting history and current results. They encourage the MD team to review differential for pediatric chest pain. Emphasize that patients with Turner syndrome have increased risk of aortic dissection. The cardiologist can also prompt the MD team to order a bedside ECG, CXR and POCUS if they have not yet been ordered.	Interpret ECG, CXR, and cardiac POCUS images. Provide brief presenting history and results to cardiology consultant. Verbalize concern for aortic dissection.	The patient’s pain is slightly improved after IV pain medication. HR and BP improve slightly (HR: 110, BP 140/85 mmHg) after IV pain medication. ECG demonstrates sinus tachycardia; CXR demonstrates widened mediastinum with prominent aortic knob; POCUS demonstrates dilated aortic root and abdominal aorta with intimal flap. If a diagnosis of aortic dissection has not been verbalized by MD team, cardiology consultant can suggest this as a possible diagnosis.
16-20 minutes		Request management recommendations for aortic dissection. Order computed tomography angiography (CTA) chest. Call cardiothoracic (CT) surgery. Start beta blocker IV infusion (esmolol infusion 50 micrograms/kilogram/minute). Continue IV opioid pain medication as needed.	The cardiology consultant can recommend the team order a CTA chest, discuss the case with CT surgery, start a beta blocker infusion and continue pain control as needed if not already performed by the MD team. Radiology calls requesting pregnancy test (HCG) and creatinine results prior to CTA.
21-25 minutes		Override request for creatinine and HCG and obtain CTA emergently without results. Admit emergently to the operating room (OR) vs. cardiac intensive care unit (CICU). Reassess need for pain medication. Update caregiver.	HR and BP improve with esmolol infusion (HR: 90, BP 110/60 mmHg). Remainder of lab results return (see Table 2).
Ideal Scenario Flow			
The participants enter the room to find an uncomfortable young female in significant pain. They place the patient on monitors, obtain access, and obtain a history from the parent. Vital signs reveal tachycardia and hypertension. With this information they develop a differential diagnosis for chest pain, recognizing that patients with Turner syndrome have increased cardiovascular risks. The team gives IV opioid pain medication with some relief and improvement in heart rate and blood pressure. The team orders appropriate labs and bedside imaging and calls cardiology to discuss the results and their concern for aortic dissection. Cardiology helps the team with management suggesting continued pain control, IV infusion of beta blocker, and STAT CTA chest and CT surgery consult. Vital signs improve with the initiation of IV infusion of beta blocker and the team admits to OR vs CICU.			
Anticipated Management Mistakes			
(1) Failure to identify and treat pain: Identifying and treating pain can be difficult but is paramount in pediatric resuscitation. If the participants don’t recognize and treat the patient’s pain, the embedded parent can emphasize the patient’s pain; the embedded RN can suggest IV opioid pain medication. (2) Failure to treat or Inappropriate treatment of blood pressure: Managing hypertension in the setting of aortic dissection is unique and requires impulse control with an IV infusion of beta-blocker. If hypertension is untreated, chest pain and tachycardia continue to worsen. If hypertension is treated with hydralazine or nicardipine prior to beta blocker, blood pressure improves (to BP 135/70 mmHg), but heart rate increases (by 10 points) and chest pain worsen. (3) Failure to recognize aortic dissection: Aortic dissection in a pediatric patient is extremely rare and therefore pediatric providers will most likely not be familiar with the CXR, ECG, POCUS, or lab findings suggestive of aortic dissection. If abnormalities are not noted, embedded RN or instructor can suggest abnormalities “This part of the ultrasound (or CXR) looks different to me."			
Other Actions (The following are not management mistakes, but would not be considered helpful in the treatment of aortic dissection)			
If they give fluid bolus: no improvement in HR; no significant change in the clinical picture. If they give anxiolytic benzodiazepine: mild improvement in HR (drop by 10), mild improvement in anxiety. If they give nitroglycerine: headache, a slight drop in BP (drop systolic by 8 mmHg, diastolic by 5 mmHg), no improvement in chest pain or tachycardia.			

Appendix B: visual cues

**Table 2:** Laboratory Results This visual cue can be used to communicate laboratory results to participants during the simulation. pCO2: Partial Pressure Carbon Dioxide; HCO3-: Bicarbonate; Hgb: Hemoglobin; Na+: Sodium; iCa+: Ionized Calcium; mmHg: Millimeters of Mercury; mEq/L: Milliequivalents per Liter; g/dL: Grams per Deciliter; mmol/L: Millimoles per Liter; mg/dL: Milligrams per Deciliter; 10^9^/L: 1,000,000,000 per Liter; ng/mL: Nanograms per Milliliter

Laboratory Results				
Blood Gas Values	Test Value		Reference Range	
pH	7.45		7.32 – 7.42	
pCO2	35	mmHg	40 – 50	mmHg
HCO3-	22	mEq/L	22 – 29	mEq/L
Hgb	10.5	g/dL		g/dL
Na+	140	mmol/L	125 – 155	mmol/L
iCa+	1.2	mmol/L	0.8 – 1.6	mmol/L
Glucose	140	mg/dL	50 – 500	mg/dL
Complete Blood Count	Test Value		Reference Range	
White Blood Cells	14	10^9^/L	5 – 10	10^9^/L
Hemoglobin	10.5	g/dL	11 – 14	g/dL
Platelets	250	10^9^/L	150 – 400	10^9^/L
Basic Metabolic Panel	Test Value		Reference Range	
Sodium	140	mmol/L	135 – 145	mmol/L
Potassium	4.2	mmol/L	3.5 – 5	mmol/L
Chloride	105	mmol/L	95 – 105	mmol/L
Bicarbonate	20	mmol/L	23 – 29	mmol/L
Creatinine	0.8	mg/dL	0.6 – 1.2	mg/dL
Blood Urea Nitrogen	16	mg/dL	5 – 20	mg/dL
Glucose	170	mg/dL	70 – 100	mg/dL
Other Test Values	Test Value		Reference Range	
D-dimer	500	ng/mL	< 500	ng/mL
Troponin	0.02	ng/mL	< 0.04	ng/mL

Appendix C: critical action checklist

**Table 3:** Critical Action Checklist PALS: Pediatric Advanced Life Support; SAMPLE History: Signs & Symptoms, Allergies, Medications, Past Medical History, Last Menstrual Cycle / Last Meal, Events Leading Up To Presentation; POC: Point-of-Care; VBG: Venous Blood Gas; CBC: Complete Blood Count; BMP: Basic Metabolic Panel

Critical Action Checklist	
	Demonstrates primary and secondary patient assessments as per Pediatric Advanced Life Support (PALS) training. Place the patient on monitors. Establish two wide bore vascular access points.
	Collect a SAMPLE history (PALS) to reveal history of Turner syndrome. (Symptoms, Allergies, Medications, Past Medical History, Last meal or Last menstrual period, Events leading up to the presentation).
	Develop differential diagnosis for severe chest pain.
	Obtain appropriate labs: Point-of-care (POC) venous blood gas (VBG) with electrolytes, D-dimer, complete blood count (CBC), basic metabolic panel (BMP), troponin.
	Order and interpret bedside chest radiograph.
	Order and interpret bedside electrocardiogram.
	Perform and interpret point-of-care ultrasound (cardiac views).
	Obtain cardiology consult.
	Recognize concern for aortic dissection.
	Treat hypertension with intravenous beta-blocker infusion.
	Treat pain with intravenous pain medication.
	Obtain computed tomography angiography chest.
	Emergent cardiothoracic surgery consult.

Appendix D: evaluation form

**Table 4:** Evaluation Form RN: Registered Nurse; APN: Advanced Practitioner Nurse; RT: Respiratory Therapist; PA: Physician Assistant

Evaluation Form	
Participants respond to the following statements using a five-point Likert scale: (1 = Strongly Disagree, 2 = Disagree, 3 = Neutral, 4 = Agree, and 5 = Strongly Agree)	
The case content was relevant to my clinical practice.	1 2 3 4 5
The case content was sufficiently challenging.	1 2 3 4 5
In the simulation, I was placed in a role consistent with my professional setting.	1 2 3 4 5
In the debriefing, I felt learners had opportunities to speak and were listened to.	1 2 3 4 5
In the debriefing, medical issues were addressed.	1 2 3 4 5
In the debriefing, teamwork and communication issues were addressed.	1 2 3 4 5
In the debriefing, teamwork and communication issues were addressed.	1 2 3 4 5
Please rate the teaching effectiveness of your instructor(s) for this specific training session.	
Instructor:	excellent, good, fair, poor
Instructor:	excellent, good, fair, poor
Instructor:	excellent, good, fair, poor
Instructor:	excellent, good, fair, poor
What is your provider type: medical student, resident, fellow, attending, RN, APN, RT, Paramedic, PA, PA student, pharmacist, other	
Comments: (What can we do better? What worked well? Suggestions?)	
